# Supplementary material for: Modeling glioblastoma heterogeneity as a dynamic network of cell states
Source: Mol Syst Biol. 2021 Sep 16;17(9):e10105. doi: 10.15252/msb.202010105 (PMC8444284; doi:10.15252/msb.202010105)
Supplement: Supplementary file 5 — Source Data for Figure 3 [file MSB-17-e10105-s001.zip › Figure3A_sourcedata/GSEA_3065/hallmarks_state5.GseaPreranked.1623416302988/HALLMARK_ESTROGEN_RESPONSE_LATE.html]

Details for gene set HALLMARK\_ESTROGEN\_RESPONSE\_LATE[GSEA]

|  || Dataset | state5 |
| Phenotype | NoPhenotypeAvailable |
| Upregulated in class | na\_neg |
| GeneSet | HALLMARK\_ESTROGEN\_RESPONSE\_LATE |
| Enrichment Score (ES) | -0.2696786 |
| Normalized Enrichment Score (NES) | -0.9232349 |
| Nominal p-value | 0.63171035 |
| FDR q-value | 0.9236385 |
| FWER p-Value | 1.0 |
Table: GSEA Results Summary

  

Fig 1: Enrichment plot: HALLMARK\_ESTROGEN\_RESPONSE\_LATE      
 Profile of the Running ES Score & Positions of GeneSet Members on the Rank Ordered List

  

| PROBE | GENE SYMBOL | GENE\_TITLE | RANK IN GENE LIST | RANK METRIC SCORE | RUNNING ES | CORE ENRICHMENT || 1 | CD9 |  |  | 14 | 0.857 | 0.0430 | No |
| 2 | TFPI2 |  |  | 124 | 0.504 | 0.0570 | No |
| 3 | ABHD2 |  |  | 163 | 0.473 | 0.0773 | No |
| 4 | SLC16A1 |  |  | 186 | 0.449 | 0.0982 | No |
| 5 | EMP2 |  |  | 239 | 0.414 | 0.1138 | No |
| 6 | CD44 |  |  | 378 | 0.349 | 0.1164 | No |
| 7 | DHCR7 |  |  | 387 | 0.347 | 0.1336 | No |
| 8 | ATP2B4 |  |  | 445 | 0.326 | 0.1442 | No |
| 9 | DNAJC1 |  |  | 517 | 0.306 | 0.1521 | No |
| 10 | FGFR3 |  |  | 567 | 0.293 | 0.1618 | No |
| 11 | CPE |  |  | 624 | 0.280 | 0.1701 | No |
| 12 | IL6ST |  |  | 627 | 0.279 | 0.1844 | No |
| 13 | GLA |  |  | 654 | 0.274 | 0.1957 | No |
| 14 | CELSR2 |  |  | 750 | 0.251 | 0.1980 | No |
| 15 | PRSS23 |  |  | 831 | 0.235 | 0.2013 | No |
| 16 | PLXNB1 |  |  | 856 | 0.232 | 0.2106 | No |
| 17 | AMFR |  |  | 952 | 0.218 | 0.2113 | No |
| 18 | SLC26A2 |  |  | 1058 | 0.204 | 0.2101 | No |
| 19 | ELOVL5 |  |  | 1320 | 0.169 | 0.1895 | No |
| 20 | FOS |  |  | 1412 | 0.161 | 0.1876 | No |
| 21 | XBP1 |  |  | 1564 | 0.145 | 0.1781 | No |
| 22 | SEMA3B |  |  | 1604 | 0.141 | 0.1811 | No |
| 23 | SLC2A8 |  |  | 1811 | 0.126 | 0.1644 | No |
| 24 | PERP |  |  | 1950 | 0.115 | 0.1549 | No |
| 25 | FLNB |  |  | 2024 | 0.110 | 0.1524 | No |
| 26 | SLC1A4 |  |  | 2049 | 0.108 | 0.1553 | No |
| 27 | MEST |  |  | 2059 | 0.108 | 0.1599 | No |
| 28 | CXCL12 |  |  | 2165 | 0.101 | 0.1533 | No |
| 29 | WFS1 |  |  | 2346 | 0.089 | 0.1377 | No |
| 30 | METTL3 |  |  | 2364 | 0.088 | 0.1403 | No |
| 31 | MDK |  |  | 2458 | 0.082 | 0.1341 | No |
| 32 | SLC29A1 |  |  | 2508 | 0.079 | 0.1327 | No |
| 33 | AFF1 |  |  | 2529 | 0.077 | 0.1345 | No |
| 34 | RNASEH2A |  |  | 2560 | 0.076 | 0.1350 | No |
| 35 | SLC7A5 |  |  | 2658 | 0.071 | 0.1278 | No |
| 36 | ABCA3 |  |  | 2662 | 0.070 | 0.1311 | No |
| 37 | SCARB1 |  |  | 2808 | 0.062 | 0.1180 | No |
| 38 | CA12 |  |  | 2885 | 0.058 | 0.1125 | No |
| 39 | FKBP5 |  |  | 3209 | 0.041 | 0.0783 | No |
| 40 | CDC6 |  |  | 3392 | 0.032 | 0.0594 | No |
| 41 | HSPA4L |  |  | 3471 | 0.028 | 0.0521 | No |
| 42 | GALE |  |  | 3486 | 0.028 | 0.0520 | No |
| 43 | OLFM1 |  |  | 3559 | 0.025 | 0.0452 | No |
| 44 | TIAM1 |  |  | 3782 | 0.016 | 0.0210 | No |
| 45 | TSPAN13 |  |  | 3913 | 0.011 | 0.0070 | No |
| 46 | MAPT |  |  | 4051 | 0.005 | -0.0082 | No |
| 47 | TPBG |  |  | 4075 | 0.004 | -0.0106 | No |
| 48 | DLG5 |  |  | 4103 | 0.003 | -0.0135 | No |
| 49 | UNC13B |  |  | 4114 | 0.003 | -0.0144 | No |
| 50 | FAM102A |  |  | 4131 | 0.002 | -0.0161 | No |
| 51 | HOMER2 |  |  | 4586 | -0.018 | -0.0663 | No |
| 52 | TST |  |  | 4856 | -0.028 | -0.0952 | No |
| 53 | GINS2 |  |  | 5024 | -0.034 | -0.1122 | No |
| 54 | FARP1 |  |  | 5041 | -0.034 | -0.1123 | No |
| 55 | TOB1 |  |  | 5047 | -0.035 | -0.1110 | No |
| 56 | SIAH2 |  |  | 5063 | -0.035 | -0.1109 | No |
| 57 | RPS6KA2 |  |  | 5067 | -0.035 | -0.1094 | No |
| 58 | CA2 |  |  | 5139 | -0.038 | -0.1154 | No |
| 59 | NCOR2 |  |  | 5345 | -0.047 | -0.1360 | No |
| 60 | TOP2A |  |  | 5722 | -0.063 | -0.1751 | No |
| 61 | ISG20 |  |  | 5757 | -0.064 | -0.1756 | No |
| 62 | PDLIM3 |  |  | 5938 | -0.071 | -0.1922 | No |
| 63 | NRIP1 |  |  | 5948 | -0.072 | -0.1894 | No |
| 64 | ADD3 |  |  | 5984 | -0.073 | -0.1896 | No |
| 65 | TFAP2C |  |  | 6012 | -0.075 | -0.1887 | No |
| 66 | NAB2 |  |  | 6103 | -0.080 | -0.1947 | No |
| 67 | PDCD4 |  |  | 6228 | -0.086 | -0.2042 | No |
| 68 | SLC9A3R1 |  |  | 6276 | -0.089 | -0.2048 | No |
| 69 | ITPK1 |  |  | 6350 | -0.093 | -0.2083 | No |
| 70 | PPIF |  |  | 6616 | -0.109 | -0.2324 | No |
| 71 | UGDH |  |  | 6647 | -0.111 | -0.2301 | No |
| 72 | JAK1 |  |  | 6893 | -0.128 | -0.2510 | No |
| 73 | CHPT1 |  |  | 6977 | -0.135 | -0.2533 | No |
| 74 | CDC20 |  |  | 6985 | -0.136 | -0.2470 | No |
| 75 | RBBP8 |  |  | 7099 | -0.145 | -0.2522 | No |
| 76 | IMPA2 |  |  | 7147 | -0.150 | -0.2496 | No |
| 77 | FKBP4 |  |  | 7326 | -0.170 | -0.2608 | Yes |
| 78 | ID2 |  |  | 7333 | -0.171 | -0.2526 | Yes |
| 79 | NBL1 |  |  | 7402 | -0.181 | -0.2508 | Yes |
| 80 | BAG1 |  |  | 7474 | -0.189 | -0.2490 | Yes |
| 81 | MYOF |  |  | 7523 | -0.194 | -0.2443 | Yes |
| 82 | CAV1 |  |  | 7575 | -0.200 | -0.2396 | Yes |
| 83 | BTG3 |  |  | 7602 | -0.204 | -0.2319 | Yes |
| 84 | PRKAR2B |  |  | 7651 | -0.211 | -0.2263 | Yes |
| 85 | NXT1 |  |  | 7688 | -0.217 | -0.2191 | Yes |
| 86 | DCXR |  |  | 7713 | -0.220 | -0.2104 | Yes |
| 87 | MOCS2 |  |  | 7848 | -0.243 | -0.2128 | Yes |
| 88 | FDFT1 |  |  | 7852 | -0.244 | -0.2005 | Yes |
| 89 | RABEP1 |  |  | 7941 | -0.264 | -0.1967 | Yes |
| 90 | HPRT1 |  |  | 7974 | -0.271 | -0.1862 | Yes |
| 91 | SNX10 |  |  | 7992 | -0.274 | -0.1739 | Yes |
| 92 | BLVRB |  |  | 8154 | -0.309 | -0.1759 | Yes |
| 93 | ALDH3A2 |  |  | 8272 | -0.346 | -0.1711 | Yes |
| 94 | DYNLT3 |  |  | 8282 | -0.349 | -0.1540 | Yes |
| 95 | PCP4 |  |  | 8304 | -0.356 | -0.1378 | Yes |
| 96 | CCND1 |  |  | 8346 | -0.368 | -0.1233 | Yes |
| 97 | ARL3 |  |  | 8412 | -0.391 | -0.1103 | Yes |
| 98 | CKB |  |  | 8448 | -0.407 | -0.0930 | Yes |
| 99 | IDH2 |  |  | 8498 | -0.434 | -0.0760 | Yes |
| 100 | ETFB |  |  | 8610 | -0.499 | -0.0625 | Yes |
| 101 | COX6C |  |  | 8777 | -0.633 | -0.0483 | Yes |
| 102 | FABP5 |  |  | 8829 | -0.684 | -0.0184 | Yes |
| 103 | RAB31 |  |  | 8831 | -0.687 | 0.0172 | Yes |
Table: GSEA details [plain text format]

  

Fig 2: HALLMARK\_ESTROGEN\_RESPONSE\_LATE: Random ES distribution      
 Gene set null distribution of ES for **HALLMARK\_ESTROGEN\_RESPONSE\_LATE**
